# Production of Prodiginines Is Part of a Programmed Cell Death Process in *Streptomyces coelicolor*

**DOI:** 10.3389/fmicb.2018.01742

**Published:** 2018-08-06

**Authors:** Elodie Tenconi, Matthew F. Traxler, Charline Hoebreck, Gilles P. van Wezel, Sébastien Rigali

**Affiliations:** ^1^InBioS - Centre for Protein Engineering, Institut de Chimie B6a, University of Liège, Liège, Belgium; ^2^Department of Plant and Microbial Biology, University of California, Berkeley, Berkeley, CA, United States; ^3^Molecular Biotechnology, Institute of Biology Leiden, Leiden University, Leiden, Netherlands

**Keywords:** antitumor antibiotics, secondary metabolites, DNA-damaging agents, cell death and differentiation, bacterial development, confocal laser microscopy

## Abstract

Actinobacteria are prolific producers of antitumor antibiotics with antiproliferative activity, but why these bacteria synthetize metabolites with this bioactivity has so far remained a mystery. In this work we raised the hypothesis that under certain circumstances, production of antiproliferative agents could be part of a genetically programmed death of the producing organism. While programmed cell death (PCD) has been well documented when *Streptomyces* species switch from vegetative (nutrition) to aerial (reproduction) growth, lethal determinants are yet to be discovered. Using DNA-damaging prodiginines of *Streptomyces coelicolor* as model system, we revealed that, under certain conditions, their biosynthesis is always triggered in the dying zone of the mycelial network prior to morphological differentiation, right after an initial round of cell death. The programmed massive death round of the vegetative mycelium is absent in a prodiginine non-producer (Δ*redD* strain), and mutant complementation restored both prodiginine production and cell death. The *redD* null mutant of *S. coelicolor* also showed increased DNA, RNA, and proteins synthesis when most of the mycelium of the wild-type strain was dead when prodiginines accumulated. Moreover, addition of the prodiginine synthesis inhibitors also resulted in enhanced accumulation of viable filaments. Overall, our data enable us to propose a model where the time-space production of prodiginines is programmed to be triggered by the perception of dead cells, and their biosynthesis further amplifies the PCD process. As prodiginine production coincides with the moment *S. coelicolor* undergoes morphogenesis, the production of these lethal compounds might be used to eradicate the obsolete part of the population in order to provide nutrients for development of the survivors. Hence, next to weapons in competition between organisms or signals in inter- and intra-species communications, we propose a third role for antibiotics (in the literal meaning of the word ‘against life’) i.e., elements involved in self-toxicity in order to control cell proliferation, and/or for PCD associated with developmental processes.

## Introduction

Traditionally, bacteria were regarded as unicellular microorganisms that rapidly grow and divide via binary fission. The concept of multicellularity among prokaryotes was recognized only three decades ago, and is particularly evident in actinobacteria, cyanobacteria, and myxobacteria (Shapiro, 1998; Claessen et al., 2014). These are bacteria with a complex life cycle and morphological and chemical differentiations that are switched on in response to environmental signals. A hallmark of multicellular organisms is programmed cell death or PCD (Bayles, 2014; Claessen et al., 2014; Lyons and Kolter, 2015). In *Bacillus subtilis*, sporulation is preceded by a form of PCD known as cannibalism in a subpopulation of the biofilm, and serves to produce the building blocks required for a new round of macromolecule synthesis to sustain developmental growth (González-Pastor et al., 2003; González-Pastor, 2011). In *Myxococcus*, three subpopulations that show division of labor arise, with cells differentiating into spores or into peripheral rods, while the remaining cells undergo PCD (Nariya and Inouye, 2008). In the cyanobacterial *Anabaena*, cell death is controlled by the circadian rhythm, which implies careful programming (Lee and Rhee, 1999).

Several rounds of PCD occur as part of the complex developmental program of the mycelial *Streptomyces* (Miguélez et al., 1999, 2000; Fernández and Sánchez, 2002; Manteca et al., 2005; Filippova and Vinogradova, 2017). Streptomycetes are filamentous Gram-positive high G+C bacteria that reproduce via sporulation. Their life cycle starts with a spore that germinates to grow out and form a mycelium consisting of multinucleoid vegetative hyphae. When reproduction is required, the older mycelium is used as a substrate to form aerial hyphae, which differentiate into chains of unigenomic spores. This morphological differentiation is accompanied by chemical differentiation, whereby many natural products, including antibiotics, anticancer compounds and antifungals, are produced. The lytic degradation of the vegetative mycelium is a clear manifestation of PCD, presumably to provide the nutrients necessary to produce the reproductive aerial hyphae (Méndez et al., 1985; Miguélez et al., 1999; Manteca et al., 2005; Chater, 2006).

The molecular triggers and genetic basis for PCD in mycelial bacteria are still largely unknown. ‘Antibiotics’ should be regarded as serious candidates for eliciting PCD processes. Antibiotic resistance is indeed mandatory for antibiotic makers such as streptomycetes, which infers that their ‘weapons’ against competitors are also often active against the producing species (Tomasz, 2006; Hopwood, 2007; Alkhatib et al., 2012; Mak et al., 2014; Ogawara, 2015; Sugiyama, 2015). To survive production of their natural products, *Streptomyces* possess self-defense mechanisms identical to those developed and acquired by human and animal pathogens (D’Costa et al., 2006; Wright, 2012; Surette and Wright, 2017). Self-toxicity - and therefore self-resistance - extends well beyond antibacterial agents; for example, anticancer molecules damaging DNA are also deadly to the producer (Galm et al., 2005). Mechanisms of resistance to antitumor antibiotics that destroy DNA have been identified in almost all producers and include (i) drug sequestration, (ii) drug inactivation (modification or destruction), (iii) drug efflux, and (iv) target repair or protection (Tenconi and Rigali, 2018).

Amongst the *Streptomyces* molecules currently explored in human disease therapy, the tripyrrole red-colored prodiginines or prodigiosin-like pigments (PdG) have gained interest due to their promising antitumor, immunosuppressive and anti-inflammatory properties (Montaner and Pérez-Tomás, 2003; Pérez-Tomás et al., 2003; Williamson et al., 2007; Pérez-Tomás and Viñas, 2010). Their topoisomerase-inhibiting activity by intercalating DNA and the many other ways they are harmful to cell components (Williamson et al., 2007; Darshan and Manonmani, 2016; Yang et al., 2017; Zhang et al., 2017) explains why prodiginines are toxic to so many different organisms and also display antimalarial, anthelmintic, antifungal, and antibacterial activities (Stankovic et al., 2014). Such a broad spectrum of activity suggests that the PdG-producing species might be sensitive to their own molecules. Indeed, in *Streptomyces coelicolor* the onset of expression of the *red* cluster (encoding the PdG biosynthetic pathway) coincides with the entrance of this species into a period of growth cessation – the so-called transition phase (Takano et al., 1992; Bibb, 1996; White and Bibb, 1997; Sun et al., 1999; Huang et al., 2001; Zhou et al., 2005; Williamson et al., 2006). In addition, prodiginine production coincides with a round of massive cell death in the course of which the *Streptomyces* multicellular filamentous network undergoes drastic morphological changes associated with the sporulation process. Time-space monitoring of *redD* expression, the activator of PdGs *red* biosynthetic genes, is also confined to aging, lysed filaments (Sun et al., 1999). Moreover, prodigiosin-like pigments are induced by stressing culture conditions like excess of metal ions and pH shock (Mo et al., 2013; Morgenstern et al., 2015), co-cultivation with competing microorganisms (Luti and Mavituna, 2011b; Schäberle et al., 2014), and feeding the medium with dead bacterial cells (Luti and Mavituna, 2011a). Finally, all other DNA-damaging agents have been shown to be toxic for the producing actinobacterial species (Tenconi and Rigali, 2018). The absence of resistance genes within the *red* cluster thus further supports the hypothesis that production of these compounds might be harshly destructive to *S. coelicolor*.

In the light of the known high cytotoxicity of prodiginines, it is remarkable that the DNA-damaging PdGs are not secreted by *S. coelicolor*, but rather accumulate internally (in the cytoplasm and within the membranes and cell wall), right at the time of growth cessation. The seemingly suicidal and at the same time well-programmed production of prodiginines suggests that these molecules may play a role in the control and/or progress of PCD. An advantage of using PdGs as our model to correlate toxin biosynthesis to PCD is that the timing and localization of their production is easily monitored *in situ* due to their red autofluorescence (Tenconi et al., 2013). In this work we demonstrate that prodiginine production correlates to dying filaments in time and space and that absence of PdGs reduces cell death in *S. coelicolor*, resulting in hyper-accumulation of viable filaments. We propose that prodiginines are main protagonists of a PCD process in the producing organisms.

## Materials and Methods

### Strains and Culture Conditions

*Streptomyces coelicolor* M145 and its *redD* mutant M510 (Floriano and Bibb, 1996) were used as the wild-type strain and as PdGs non-producer, respectively. R2YE (Kieser et al., 2017) agar plates were used for phenotypic characterization. Where applicable, R2YE plates were covered with cellophane membranes (GE Osmonics Labstore, Ref K01CP09030). Plates were inoculated with 500 μl of a 2 × 10^7^ cfu/ml spore suspension. Where applicable, *N*-acetylglucosamine was added to a final concentration of 25 mM in R2YE plates.

### Complementation of the *redD* Mutant M510

The *S. coelicolor redD* mutant M510 was complemented by introducing plJ2587 harboring *redD* (SCO5877) with its native upstream region. A DNA fragment containing the *redD* upstream region (567 bp) was generated by PCR using primers (5′- GAATTCCCCCTGCTGCTCCAGGG -3′) and (5′- GGATCCCCCAATATGTTGATTTCCACGC -3′) with engineered EcoRI and BamHI sites, respectively, and cloned into pJET1.2 (Thermo Fisher Scientific). After sequence confirmation, the fragment was retrieved through EcoRI and BamHI restriction digest, gel purified, and cloned into an EcoRI/BamHI-linearized plJ2587 (van Wezel et al., 2000) upstream of *redD* resulting in plasmid pELT003. The complementation construct, as well as the empty plJ2587 plasmid, were introduced into the *redD* mutant through intergeneric conjugation as described previously (Tenconi et al., 2015). All thiostrepton resistant colonies transformed with pELT003 (gene *tsr* in plJ2587) presented the intracellular red pigmentation and red fluorescence associated with PdG production confirming the complementation of the *redD* mutant phenotype.

### *In situ* Visualization by Confocal Fluorescence Microscopy

Samples were prepared as described previously (Tenconi et al., 2013). Samples were examined under Leica TCS-SP2 and Leica TCS-SP5 confocal laser-scanning microscopes. SYTO9 and SYTOX stained samples were examined at a wavelength of 488 for excitation and 530 nm (green) for emission. Red autofluorescence of PdGs and propidium iodide-stained samples were examined at a wavelength of 543 nm (Leica TCS-SP2) or 568 nm (Leica TCS-SP5) for excitation and 630 nm (red) for emission as described previously (Tenconi et al., 2012). Quantitative analyses were performed employing the Leica LAS-AF image analysis program. Image processing and 3D reconstruction of *Streptomyces* filaments were performed as described previously (Tenconi et al., 2013).

### DNA, RNA, and Protein Extraction and Quantification

Total DNA, and intracellular and extracellular RNA were extracted using the phenol/chloroform/isoamyl alcohol protocol, mainly as described previously (Sambrook et al., 1989). The *S. coelicolor* mycelium (from 24, 32, 40, 48, 56, 65, and 72 h cultures) was scraped with a spatula from the R2YE agar plates covered with cellophane disks, put into 2 ml tubes and frozen at -70°C. 50 mg of mycelium were first subjected to lysozyme digestion (2 mg/ml final concentration in 2 ml of extracted buffer pH8), and then incubated with proteinase K (0.5 mg/ml; 1 h at 55°C) prior to nucleic acids extraction. DNA was removed from total nucleic acids extracted by using the kit Turbo DNA-free (Ambion). Proteins were extracted from 50 mg of mycelium as described previously (Tenconi et al., 2013) after sonication using 30 s pulse for 10 min (Bioruptor, Diagenode, Liège, Belgium) in 500 μl of extraction buffer. Protein concentration was determined by measuring absorbance at 280 nm.

## Results

### *In situ* Visualization of Prodiginine Production During the Life Cycle of *S. coelicolor*

**FIGURE 1:**
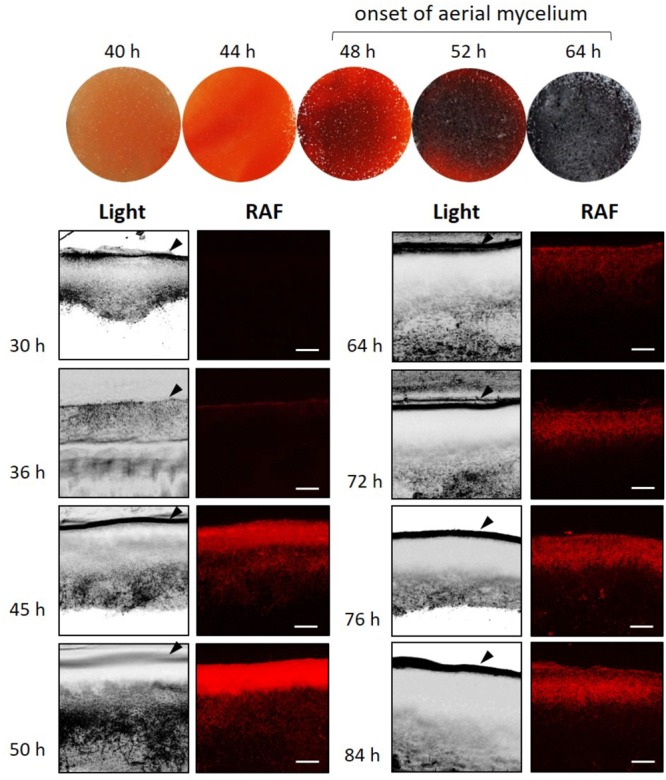
Visualization and quantification of RAF throughout the life cycle of *Streptomyces coelicolor*. *In situ* visualization of RAF by confocal microscopy in cross section of a confluent *S. coelicolor* culture on R2YE agar plates. Bars, 40 μm. Arrows indicate the surface of the agar plate cultures. Phenotypes of *S. coelicolor* grown in R2YE agar plates are shown to visualize the delay between the onset of PdG production and the onset of the morphological differentiation (white mycelium appearing on the surface of the plate).

Prodiginine (PdG) production was monitored throughout the life cycle of *S. coelicolor*, making use of their red autofluorescence (RAF) as described previously (Tenconi et al., 2013). Spores (10^7^ cfu) of *S. coelicolor* M145 were spread onto the surface of R2YE agar plates, and 0.5-mm thick slices of confluent solid cultures were collected at different time points and imaged via confocal fluorescence microscopy. *In situ* visualization of PdG production (under non-saturated excitation conditions) revealed that weak RAF appeared at the surface of the vegetative mycelium at around 36 h, and that this signal reached its maximum level at 50 h (**Figure 1**). From that time point onwards, the RAF intensity decreased abruptly to persist at approximately one third of its maximal intensity (**Figure 1** and Supplementary Figure S1). The accumulation of PdGs was therefore maximal (∼50 h) at the morphological transition phase, before the vegetative mycelium differentiates into aerial hyphae (between 50 and 64 h, **Figure 1**).

### Time-Space Correlation Between Prodiginine Production and Cell Death

**FIGURE 2:**
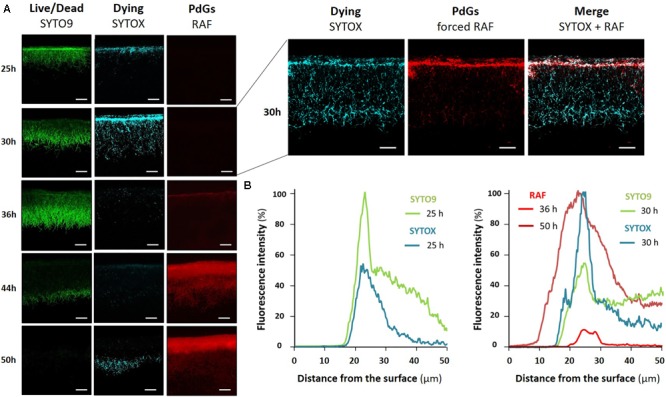
Spatio-temporal occurrence of production of PdGs and cell death in *S. coelicolor*. **(A)** Images represent cross-sections of confluent lawns of *S. coelicolor* M145 grown on R2YE agar plates. Maximal fluorescence intensity associated with PdG biosynthesis (RAF), dying filaments (SYTOX-staining, blue-colored), or live/dead filaments (SYTO9-staining, green colored), was reached after 50, 30, and 25 h of growth, respectively. The voltage on the photomultiplier tubes (PMT) for RAF was fixed to 600 V. On the right panels the PMT was increased to 680 V in a 30 h old cross section in order to visualize RAF showing early PdG production that starts from the thin band of cell death. Bars, 40 μm. **(B)** Quantification of RAF, SYTO9, and SYTOX fluorescence signals across the first 50 μm of the *S. coelicolor* culture. Note that the peak of maximum RAF, SYTO9, and SYTOX occurred in the same thin band at a distance of about 20–30 μm from the surface of the culture.

Simultaneous *in situ* quantification of RAF and dying cells revealed that the membrane-damaged filaments, which are permeable to SYTOX, reached their highest level at around 30 h of culture, about 20 h before the maximal intensity of RAF (∼50 h) (**Figures 2A,B**). This maximum of accumulation of dying filaments occurred ∼5 h after the peak of SYTO9 fluorescence (∼25 h), which, at time points prior to PdG production (see below), stains both live and membrane damaged (dying) cells (**Figures 2A,B**). Quantification of SYTO9 and SYTOX signals along the first 50 μm of the *S*. *coelicolor* culture revealed that both dyes present a peak of fluorescence at the same distance of ∼20–30 μm from the surface of the culture (**Figure 2B**). This spatial co-localization at an approximately 5 h interval suggests that dying filaments observed at 30 h emanated from those presenting the highest SYTO9 signal at 25 h. Quantification of PdGs along the same vertical axis showed that the earliest RAF signal detected at 36 h (under non-saturated excitation conditions **Figures 2A,B**) also arises at the same distance of ∼20–30 μm from the surface of the culture, right after the maximum of accumulation of dying cells (SYTOX-staining, **Figure 2**). Increasing the voltage on the photomultiplier tubes (PMT) during imaging of 30 h old samples revealed that the forced early RAF signal matched the thin 10 μm band of the culture where first dying filaments were seen (**Figure 2A**, right panel) supporting the idea that PdG biosynthesis spatially coincides with dying cells but would be posterior to the occurrence of this early cell death event.

**FIGURE 3:**
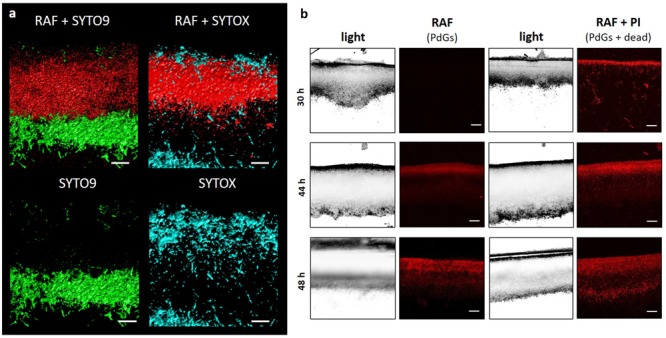
Localization of the production of prodiginines in the dying zones of the *S. coelicolor* culture. **(a)** Fluorescence confocal micrographs (3D reconstruction) of two transverse sections of an *S. coelicolor* M145 culture grown for 40 h on R2YE agar plates. Left panel: RAF (red-colored) and staining of viable filaments (green-colored, SYTO9). Right panel: RAF (red-colored) and staining of dying filaments (blue-colored, SYTOX). Bar, 20 μM. **(b)** Visualization of the red fluorescence associated with PdG production (RAF) and propidium iodide staining (PI). Bar, 20 μM.

At a later time point (40 h) qualitative analysis of PdG fluorescence and accumulation of dying cells (SYTOX stained) along the vertical axis of a confluent solid culture showed a clear correlation between RAF and the zone of cell death visible in the upper part of the culture (**Figure 3a**). Conversely, no or extremely weak RAF was seen in SYTO9 stained sections, and *vice versa* (**Figure 3a**). The fact that maximum RAF (50 h) coincided with (i) the lowest amount of SYTO9 (live and dead) staining, and (ii) maximal SYTOX (dead) staining (**Figure 3a**) further supports that PdGs might be associated with the massive round of cell death observed at the surface of the culture and preceding the morphological differentiation of *S. coelicolor*. To further demonstrate that PdGs accumulate in the dead zone at the surface of the *S. coelicolor* culture, we repeated the monitoring of dying cells using propidium iodide (PI) as alternative fluorescent dye for staining membrane-damaged filaments. PI displays maxima of excitation/emission of red fluorescence very close to the maxima of RAF of PdGs (Tenconi et al., 2013). Monitoring PI fluorescence will thus also reveal red fluorescence when PdGs are not produced. At 30 h of growth, prior to PdG production, PI display fluorescence at the upper part of the culture (**Figure 3b**) with a spatial pattern similar to the one observed with SYTOX staining (**Figure 2A**). At later time points, when PdGs are produced, the fluorescence of PI in the upper part of the culture (**Figure 3b**) is observed in the same zone as where RAF associated with PdGs was observed (**Figures 1, 2**), the fluorescence signal being increased when both RAF and PI were recorded. That RAF recorded together with PI fluorescence staining dying filaments display similar spatio-temporal patterns as those recorded for RAF alone further supports that PdGs accumulate in the dead zone at the surface of the culture.

### Monitoring of PdGs Biosynthesis at Filament Size Scale

**FIGURE 4:**
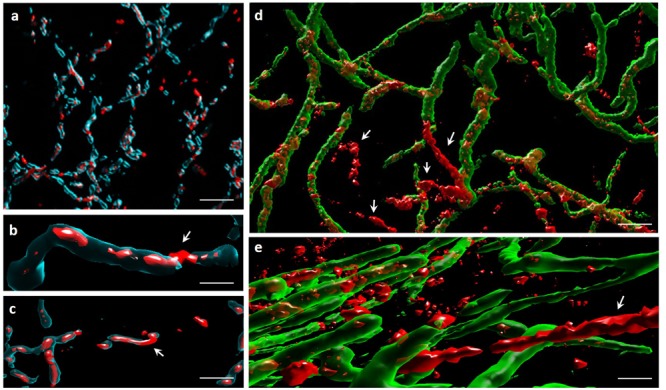
Monitoring of PdG production at the filament size scale in *S. coelicolor*. **(a–c)** Filaments of *S. coelicolor* M145 inoculated on R2YE plates displaying both RAF and SYTOX (blue) staining. White arrows point to portion of filaments not stained with SYTOX and with abundant PdG production visualized by RAF. Bars, 10 μm **(a)**, 2 μm **(b)**, and 5 μm **(c)**. **(d,e)** Filaments of *S. coelicolor* M145 inoculated on R2YE plates displaying both RAF and SYTO9 (green) staining. White arrows point to ‘ghost filaments’ with abundant production of PdGs and therefore not stained with SYTO9. Bars, 2 μm **(d)**, and 1 μm **(e)**.

We attempted to monitor PdG production at the filament size scale in order to assess if they are produced by dying or living filaments. Repetitive assays revealed that, at time points where *S. coelicolor* abundantly produces PdGs (from 40 h onwards), SYTO9 and SYTOX hardly stained any of the filaments that display RAF. Visualization of filaments that display both RAF, and SYTOX or SYTO9 fluorescence was only possible at lower mycelial density (below the zone of high fluorescence signals in the first 50–60 μm), or at the brief moment in the life cycle (30 h) when the level of PdGs is still low inside the filaments. Specimens where we could observe intracellular PdG production inside filaments stained with SYTO9 or SYTOX are presented at **Figure 4**. However, once PdG accumulated above a certain level inside the filaments, SYTOX and SYTO9 failed to stain, leading to partial or complete ‘ghost’ filaments that could only be visualized by RAF (**Figure 4**).

This inhibition of SYTO9 and SYTOX staining by PdGs is most likely the result of the DNA-intercalation ability of PdGs and/or because of the PdGs-induced destruction of nucleic acids (see below **Figure 6**). The observed lack of staining with SYTOX or SYTO9 once the RAF signal is high, and therefore when PdG production abundantly accumulated, led us to hypothesize that these DNA-damaging metabolites that remain intracellular, may be involved in the destruction of the DNA in the vegetative mycelium of *S. coelicolor*. However, RAF was more frequently observed inside filaments stained with SYTO9 compared to filaments stained with SYTOX, suggesting that their production occurs in living filaments, causing cell death most likely by damaging DNA, therefore causing SYTOX staining more casual once PdGs were produced. Alternatively, the rarely observed co-staining of PdGs-SYTO9 and PdGs-SYTOX at the filament scale could be attributed to the DNA-bound prodiginines that could sterically hinder SYTOX or SYTO9 dyes to access DNA.

### PdGs Have Anti-proliferative Activity

**FIGURE 5:**
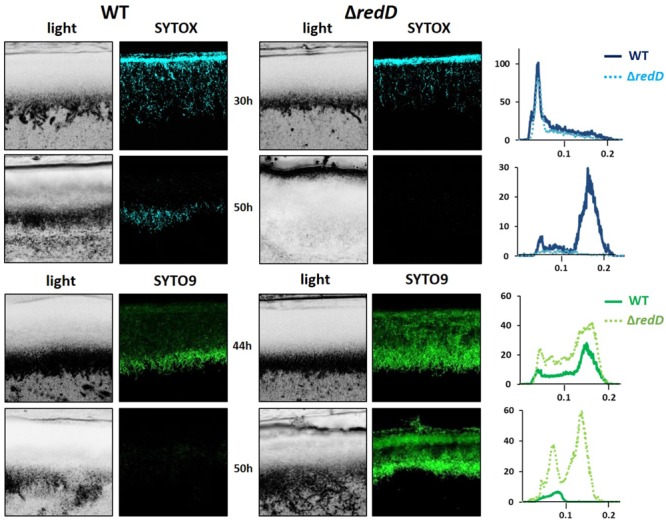
Prodiginine biosynthesis causes massive loss of viable filaments. SYTOX (blue) and SYTO9 (green) staining of cross sections of culture of *S. coelicolor* M145 (wild-type, WT) and its *redD* null mutant inoculated in R2YE agar plates. Bars, 40 μm. Note that (i) the PCD round prior to PdG production also occurs at 30 h in the *redD* null mutant of *S. coelicolor*, and (ii) the higher accumulation of viable filaments (only stained by SYTO9) in the *redD* mutant. Quantification of the SYTOX and SYTO9 fluorescence signals are displayed in plots next to the microscopy pictures.

The observed lack of staining with SYTOX or SYTO9 once the RAF signal is high, and therefore when PdG production abundantly accumulated, led us to hypothesize that these DNA-damaging metabolites that remain intracellular, may be involved in the destruction of the DNA in the vegetative mycelium of *S. coelicolor*. To investigate this, we first compared the accumulation of viable and dying filaments between *S. coelicolor* M145 and its *redD* null mutant M510, which fails to produce PdGs (**Figure 5**). The *redD* null mutant also displayed a band of dying hyphae at the upper surface of the culture at 30 h (**Figure 5**), which may be seen as an argument that the production of PdGs is a consequence, and not the cause, of the onset of cell death. However, at 50 h of growth, which corresponds to the time point of maximal RAF intensity in the wild-type strain (**Figures 1, 2**), the PdG-non-producing strain M510 still displayed many SYTO9-stained cells, while the parental strain M145 showed only very weak staining along the vertical axis of the culture (**Figure 5**). Importantly, we did not observe any fluorescence when SYTOX was added after 50 h of growth in the *redD* null mutant (**Figure 5**), suggesting that all filaments stained by SYTO9 were viable. The absence of SYTO9 and SYTOX staining in the zone of the culture that displayed maximal RAF suggests that PdGs had caused widespread DNA destruction within the vegetative mycelium preventing the DNA-interacting commercial dyes to bind their molecular target. Complementation of the *redD* mutant restored PdG production and resulted in the subsequent massive loss of filaments stained with SYTO9 (Supplementary Figure S2) demonstrating that the absence of the massive death round in the *redD* mutant was caused by the loss of PdG production.

**FIGURE 6:**
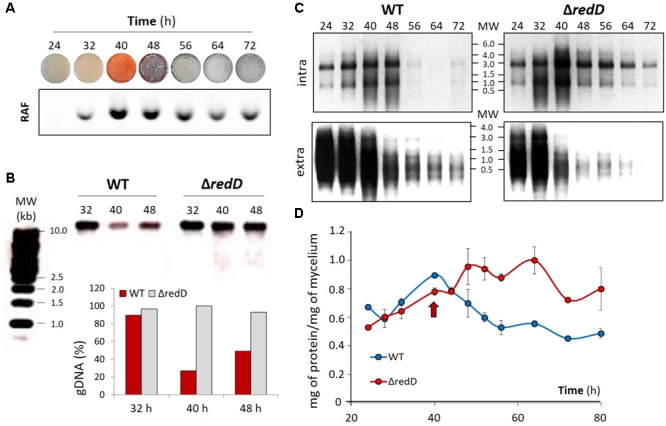
Effect of the deletion of *redD* on DNA, RNA, and protein synthesis. **(A)** PdG production throughout the life cycle of *S. coelicolor*. RAF from mycelium extracts of *S. coelicolor* grown on the R2YE medium covered with cellophane disks. Extracts were deposited in an agarose gel and RAF was monitored after migration as described previously (56). **(B)** Ethidium bromide stained agarose gels and quantification showing the amount of genomic DNA extracted from 50 mg of mycelium of *S. coelicolor* M145 (WT) and its *redD* null mutant (Δ*redD*). 100% refers to the maximum of genomic DNA collected, i.e., in the *redD* mutant at 40 h. **(C)** Ethidium bromide stained agarose gels showing intracellular (top panels) and extracellular (bottom panels) RNA extracted from *S. coelicolor* M145 (WT) and its *redD* null mutant (Δ*redD*). **(D)** Quantification of the total protein content in crude extracts of mycelia of *S. coelicolor* WT (M145) and its *redD* null mutant. The red arrow indicates the timing of maximal PdGs biosynthesis.

Additionally, we monitored and compared other markers of viability and metabolic activity between the wild-type strain M145 and the *redD* mutant, namely DNA, RNA, and protein synthesis (**Figure 6**). For this purpose, R2YE agar plates were covered with cellophane disks prior to inoculation with spores of either *S. coelicolor* M145 or its *redD* mutant M510, to allow collection of the biomass from the spent agar. The presence of the membranes on the top of the plates caused accelerated production of PdGs, with a peak in RAF intensity 10 h earlier than observed in plates not covered with cellophane disks (40 h instead of 50 h) (**Figure 6A** and Supplementary Figure S1). The genomic DNA collected prior (32 h), and during (40 and 58 h) PdG synthesis revealed less accumulation of DNA in the wild-type strain *S*. *coelicolor* once PdG are being produced while the amount of DNA remained constant in the *redD* mutant M510 (**Figure 6B**). Similarly, analysis of total RNA isolated from the wild-type strain *S*. *coelicolor* and its red mutant also revealed a drop in the accumulation of 16S and 23S rRNA in the wild-type strain right after the peak of PdG biosynthesis (**Figure 6C**). In contrast, after that time point, rRNA levels remained significantly higher in the *redD* mutant as compared to the parental strain (**Figure 6C**). Interestingly, the amount of RNA collected from the washed mycelium was instead significantly higher in the wild-type strain than in the *redD* mutant, suggesting more intense lysis of the parent and consequential accumulation of RNA outside the filaments (**Figure 6C**). Assessment of the total protein content of the intracellular crude extracts also revealed distinct profiles between the two strains, with the *redD* mutant displaying higher amounts throughout the time course as compared to the parental strain, which showed a drastic drop of protein accumulation just after the peak of RAF at 40 h (**Figure 6C**). The higher intracellular accumulation of macromolecules (DNA, RNAs, and proteins) strongly suggests that the metabolism of the PdG-non-producer remains active at stages of the life cycle where the large majority of the vegetative mycelium of the wild-type strain is encountering destructive processes.

### Inhibiting Conditions for PdG Production Reduces Cell Death

**FIGURE 7:**
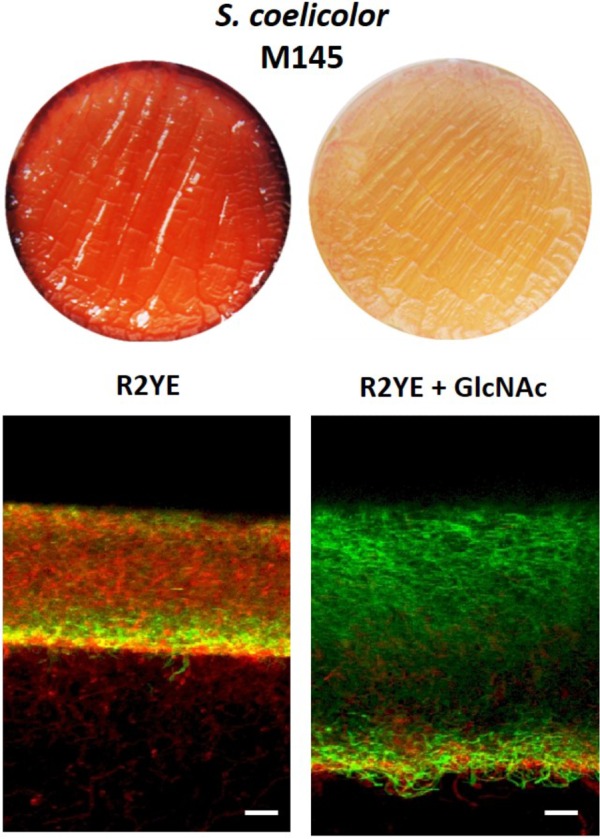
Addition of the PdG biosynthesis inhibitor GlcNAc results in accumulation of viable filaments. Effect of GlcNAc (25 mM) on SYTO9 (green) filament staining, and PdG and PI (red) fluorescence. Bars, 20 μm. *S. coelicolor* M145 was inoculated in R2YE agar plates.

To further test the idea that *S. coelicolor* itself is vulnerable to intracellular PdGs and that PdG are induced by cell death, we used a culture condition known to strongly reduce PdG production in *S. coelicolor* and assessed if it also correlates with reduced cell death and/or higher accumulation of viable filaments. 2.10^7^ spores of *S. coelicolor* M145 were used to inoculate the R2YE solid medium with or without addition of the aminosugar *N*-acetylglucosamine (GlcNAc) known to block morphogenesis at the vegetative state and PdG production in *S. coelicolor* grown on rich media (Rigali et al., 2006, 2008) (**Figure 7**). *In situ* visualization of samples of confluent solid cultures collected at 42 h revealed that the presence of the morphogenesis-blocking agent GlcNAc resulted in a massive accumulation of SYTO9 stained filaments (**Figure 7**). As GlcNAc containing samples were devoid of the red fluorescence associated with PdG production and PI staining (**Figure 7**), it suggests that all SYTO9 stained filaments were viable. In contrast, without GlcNAc, the mycelium of *S. coelicolor* displayed important red fluorescence (from both PI and PdGs) associated with dying cells and presented a much lower growth as deduced from the thickness of the mycelial culture (**Figure 7**). The same experiment was previously performed by adding to the R2YE medium other types of PdG synthesis inhibitors such as phosphorylated sugars (Tenconi et al., 2012). The addition of phosphorylated nutrients (glycerol-3-P, glucose-6-P, Frucrose-1,6-BP) inhibited PdG production and reduced/delayed cell death (Tenconi et al., 2012). Importantly, the addition of counterpart non-phosphorylated nutrients neither reduced PdG synthesis nor cell death (Tenconi et al., 2012) suggesting that the observed phenomenon with phosphorylated sugars was not due to preventing nutrient starvation. Next to our study of the phenotype of the *redD* mutant, the use of PdG synthesis inhibitors also revealed that preventing or reducing PdG synthesis results in reduced cell death and higher accumulation of viable filaments.

## Discussion

Do natural products with anti-proliferative activities, such as PdGs, participate in the PCD process of the producing microorganism? As PdGs are not secreted but accumulate internally or remain in the membrane in *S. coelicolor*, this hypothesis is directly in line with the concept of these molecules being auto-destructive rather than acting on neighboring microbes. We found that, under the tested conditions, the production of PdGs is programmed to always coincide in space with an early and limited cell death event. PdG synthesis follows in time this early cell death event and their production amplifies the phenomenon by killing the overwhelming majority of the *S. coelicolor* culture. This broad and rapid destruction of the mycelium is assisted by the PdGs as in the PdG non-producing strain (the *redD* null mutant) cell death was attenuated, and DNA, RNA, and protein synthesis continued at time points where normally these processes are abolished in the parental strain. Loss of cell death and accumulation of viable filaments were also observed when the culture medium was supplemented with the PdG production inhibitors.

### Cell Death Is the Trigger of PdG Biosynthesis

Next to phenotypic characterization of mutants, a complementary means to uncover the role(s) played by a secondary metabolite in nature is to identify signals that regulate its biosynthesis (i.e., ‘*what controls you will tell us who you really are*’). When we visualized RAF at an early time point, we found that the biosynthesis of PdGs is not triggered homogeneously but is instead spatially restricted to a very thin (<10 μM) band in the upper part of the *S. coelicolor* culture (**Figure 2**). This thin band of PdG production arises exactly where dying cells are constantly observed at the same moment of the life cycle of *S. coelicolor*. This observation corroborates previous findings that *redD* expression occurred only when a proportion of the hyphae had lysed, suggesting that transcription of *redD* is confined to aging vegetative mycelium (Sun et al., 1999). It has also been demonstrated that the production of PdGs of *S. coelicolor* is induced by the addition of dead cells in the culture medium (Luti and Mavituna, 2011a). The initial band of cell death where the production of PdGs started also corresponds to the thin band where we observed maximum SYTO9 and SYTOX staining at 25 and 30 h, respectively (**Figure 2**). PdG biosynthesis thus arises where the density of the population is maximal and therefore could be governed by a quorum sensing regulatory pathway. That synthesis of killing factors (PdGs) follows in time with the zone of the culture with the highest density of the population is most likely not just a coincidence. The high density observed at 25 h in the thin band in the upper part of the culture suggests highly active metabolism during aerobic growth with important production of reactive oxygen species (ROS) which could be the cause of the massive cell death observed in the same zone at 30 h (**Figure 2**) and proposed as triggered for the formation of the aerial mycelium of *Streptomyces* (Beites et al., 2015).

### What Would Be the Evolutionary Advantage of Producing Cytotoxic Compounds Prior to Morphogenesis?

For *Streptomyces*, it is generally accepted that the death of the vegetative mycelium provides nutrients for the advancement of later stages of the life cycle, i.e., the erection of aerial hyphae and their subsequent maturation into spore chains (Méndez et al., 1985; Miguélez et al., 1999, 2000; Chater, 2006). However, we note that there is a current paucity of data that actually support this claim. At the same time, the toxicity of PdGs might also prevent scavenging of these newly liberated nutrients by other bacteria and/or fungi. The model above (i.e., chemical protection of its own food reservoir) is appealing as it rationalizes why a PCD event might precede morphogenic development and why a small molecule that is also toxic to other organisms might be an ideal mediator of this process. In this case, the killing agent is a structurally complex compound whose synthesis requires a large cluster of 23 genes. Making PdGs might seem an expensive strategy to participate in a cell death process. But making a complex natural product is only ‘expensive’ if the evolutionary pay-off for making it is modest. However, if the pay-off is large, then it is a favorable strategy. So, for the PdGs if the pay-off is ensuring its own food reservoir during the sporulation process, then it might not be expensive at all.

### Specific and Controlled Suicide

A microorganism that triggers its own cell death in a controlled manner has to make sure that (i) not all cells are killed by the process and (ii) it has to sense that the killing molecule is ‘home made.’ In other words, the compartments close to the future site of development have to undergo cell death in a controlled manner. It is important to note that the production of PdGs is indeed well controlled, and strictly correlates to the so-called transition phase in submerged cultures, and to the phase immediately preceding the onset of morphological development in solid-grown cultures (Takano et al., 1992; Bibb, 1996; Sun et al., 1999). This strongly suggests that the cell death caused by the PdGs is indeed programmed, in other words represents PCD in the true sense, as a way to eradicate the old mycelial biomass for the next growth phase, namely the aerial hyphae and spores. One could easily imagine that the physiological reaction to sensing the presence of a toxic compound must be very different if the molecule originates from competitors that share the same environmental niche or, instead, if the molecule is self-made. This also explains why microorganisms that undergo PCD to sustain metamorphosis would use their own molecule for signaling the timing of differentiation. If they would all use the same trigger their development would be synchronized, while their fitness to the environmental is different. Similarly, plants all have their own cocktail of hormones and physico-chemical parameters for inducing flowering. In addition, having a killing system not ‘universal’ (conserved molecule or mechanisms in all actinobacteria) allows also having its own mechanism of resistance [see examples of specific resistance to enediyene, daunorubicin and doxorubicin, mitomycins, bleomycins and other DNA-damaging antibiotics produced by Actinobacteria (Tenconi and Rigali, 2018)]. Therefore, a secret code in the form of a complex natural product with resistance is required.

However, a key experimental result presented here suggests that the reality is more complicated; the *redD* null mutant (which fails to produce PdGs) still undergoes an initial, limited round of PCD. Importantly, there are also many culture conditions where *S. coelicolor* does not produce PdGs but still undergoes PCD, which means that cell death can be completed in the absence of PdGs. When PdG biosynthesis is required remains unclear, but seems inextricably linked to growth conditions where microorganisms are facing challenging conditions. Indeed, previous works both in *Streptomyces* and *Serratia* species, reported that PdG biosynthesis is associated with diverse, stress-inducing culture conditions such as UV-light exposure, oxidative stress, nutrient depletion or competition with other microorganisms (Williamson et al., 2006; Luti and Mavituna, 2011a,b; Stankovic et al., 2014). In many cases authors have postulated that the ecophysiological role of PdGs is to protect the producing strain against these various stresses (Stankovic et al., 2014). How do the cell-eradicating properties of PdGs described in this work, and those also well documented for eukaryotic cell death or apoptosis (Williamson et al., 2007), fit with the proposed protective roles in various stressful conditions? Again, when part of the colony is challenged by lethal stress conditions, inducing controlled cell death in that part of the colony would be a good strategy to spare at least part of the population. In such a scenario, at least a fraction of the population might survive for dissemination to more appropriate living conditions.

## Conclusion

The data presented here shows that PdGs are closely associated with PCD process of the producing organism. Specifically, an initial phase of PCD appears to serve as a ‘detonator’ that triggers the widespread self-killing of the *S. coelicolor* biomass through the action of the PdGs. Our work highlights for the first time genes encoding toxic determinants of the PCD phenomenon in mycelial bacteria. How exactly prodiginines are destructive to *S. coelicolor* still has to be clarified as these molecules are cytotoxic in multiple ways (Williamson et al., 2007; Darshan and Manonmani, 2016; Yang et al., 2017; Zhang et al., 2017). Providing answers to how some filaments survive the production of these lethal compounds and switch to a reproductive life-style are also key questions going forward. How much the use of cytotoxic “secondary metabolites” in PCD processes is widespread in microorganisms is difficult to estimate and this question is currently under investigation. The proposed role in PCD of antitumor antibiotics could be played by other types of antibiotics (targeting the cell wall, the translational machinery,…) which increases the possibility that the observed phenomenon is not unique to prodiginines and *S. coelicolor*. Hence, next to weapons in warfare between organisms or molecules involved in inter- and intra-species communications, our work proposes a third role for antibiortic (in the literal meaning of the word ‘against life’) that is elements required for self-toxicity in PCD events either necessary to maintain an appropriate growth balance, to cope with stress conditions, or as part of developmental programs.

## Author Contributions

ET and SR designed the experiments. ET, assisted by CH, performed the experiments. All authors interpreted the data and wrote the manuscript.

## Conflict of Interest Statement

The authors declare that the research was conducted in the absence of any commercial or financial relationships that could be construed as a potential conflict of interest.
