# Supplementary figures and images for: Production of Prodiginines Is Part of a Programmed Cell Death Process in Streptomyces coelicolor
Source: Front Microbiol. 2018 Aug 6;9:1742. doi: 10.3389/fmicb.2018.01742 (PMC6087738; doi:10.3389/fmicb.2018.01742)

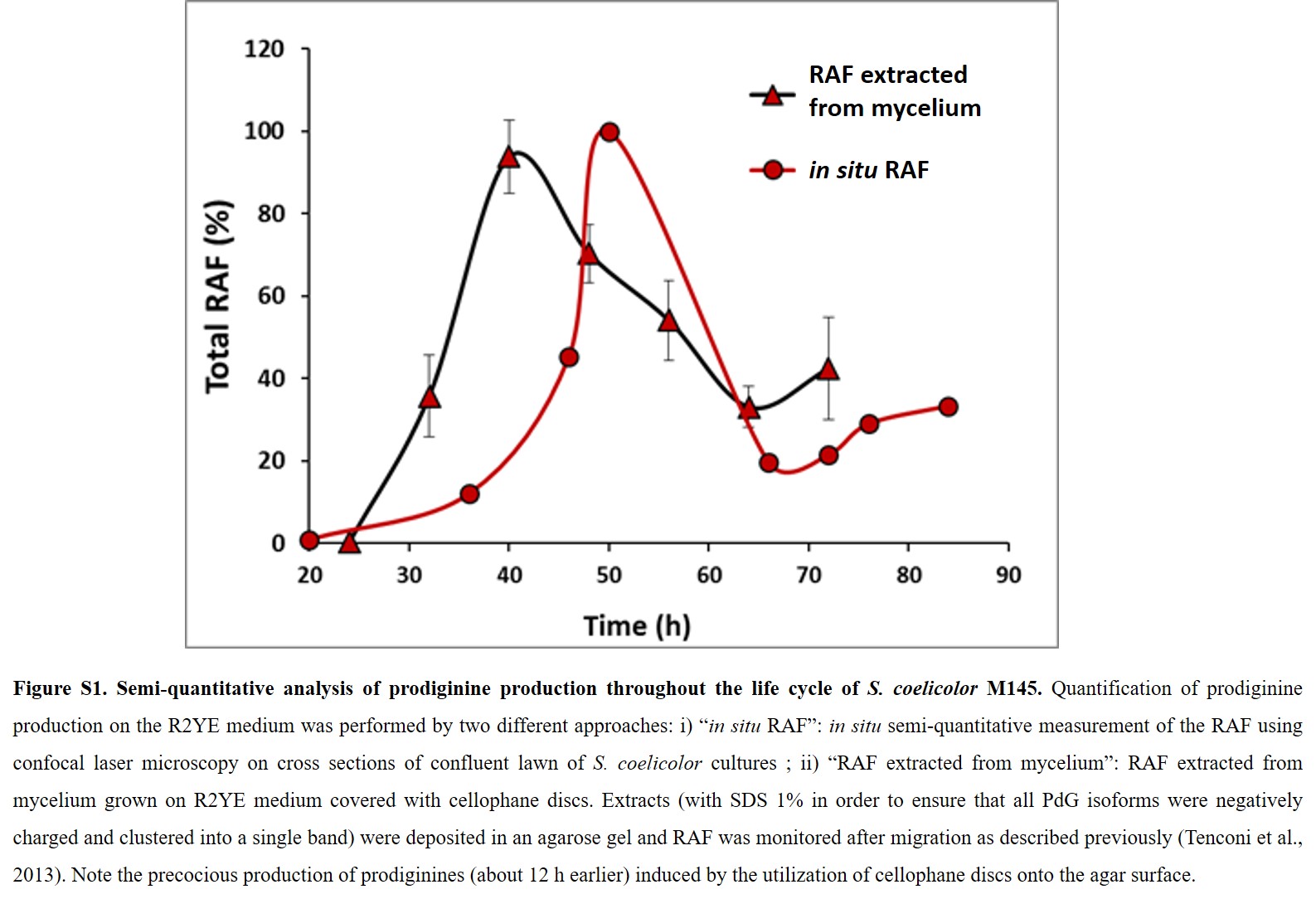

Supplement: Supplementary file 1 [file Image_1.JPEG]

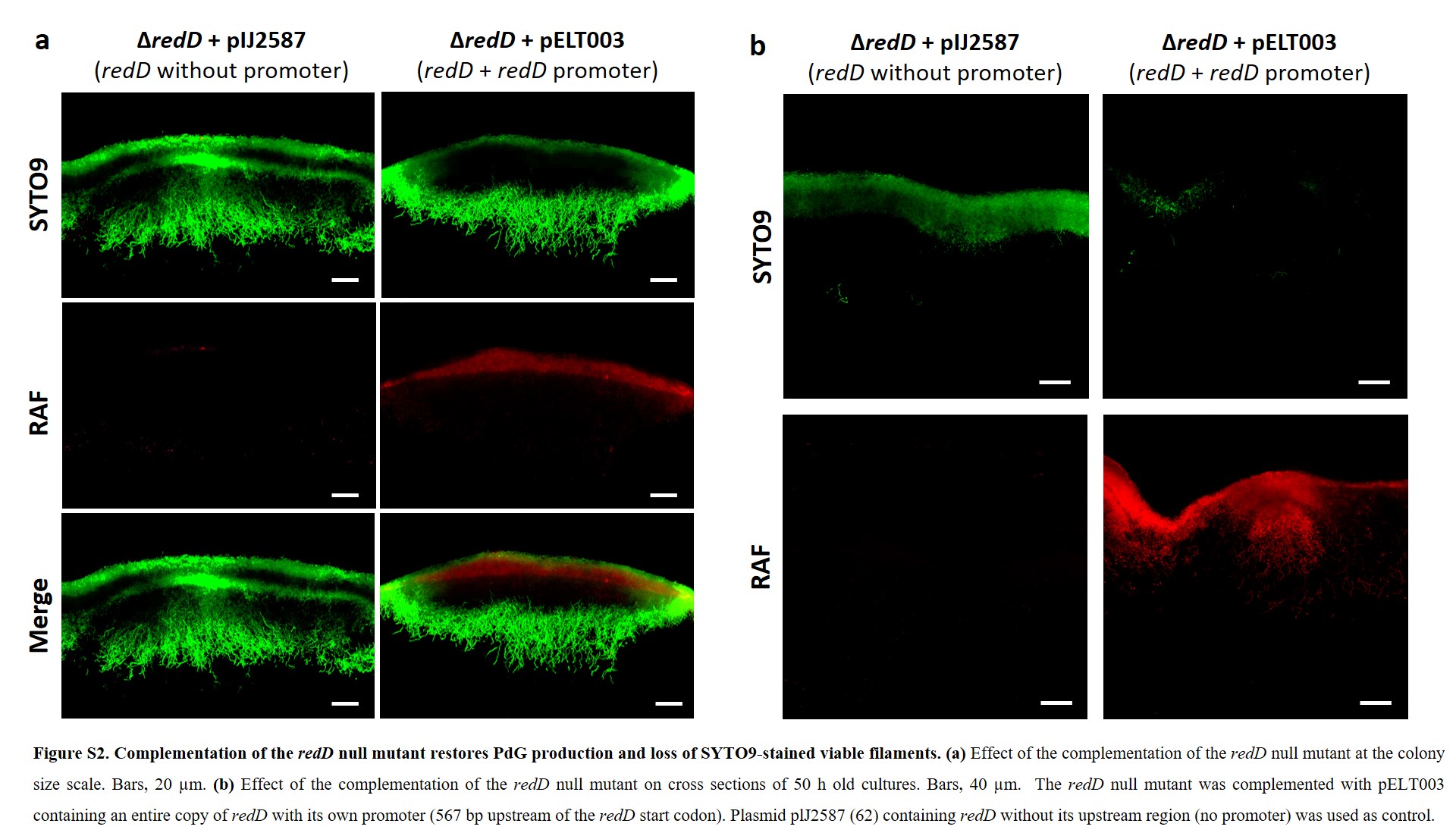

Supplement: Supplementary file 2 [file Image_2.JPEG]
